# What do the general public believe about the causes, prognosis and best management strategies for low back pain? A cross-sectional study

**DOI:** 10.1186/s12889-021-10664-5

**Published:** 2021-04-08

**Authors:** Amanda Hall, Danielle Coombs, Helen Richmond, Krystal Bursey, Brad Furlong, Rebecca Lawrence, Steven J. Kamper

**Affiliations:** 1 Primary Healthcare Research Unit, Memorial University of Newfoundland, 300 Prince Philip Dr, St. John’s, Newfoundland and Labrador NL A1B 3V6 Canada; 2 Institute for Musculoskeletal Health, University of Sydney, Sydney, Australia; 3 School of Health Sciences, Faculty of Medicine and Health, University of Sydney, Sydney, Australia; 4 Nepean Blue Mountains Local Health District, Penrith, Australia

**Keywords:** Low back pain, Back beliefs, Cross-sectional, General public

## Abstract

**Background:**

Low back pain (LBP) is one of the most common reasons for seeking health care and is costly to the health care system. Recent evidence has shown that LBP care provided by many providers is divergent from guidelines and one reason may be patient’s beliefs and expectations about treatment. Thus, examining the nature of patient beliefs and expectations regarding low back pain treatment will help coordinate efforts to improve consistency and quality of care.

**Methods:**

This study was a cross-sectional population-based survey of adults living in Newfoundland, Canada. The survey included demographic information (e.g. age, gender, back pain status and care seeking behaviors) and assessed outcomes related to beliefs about the inevitable consequences of back pain with the validated back beliefs questionnaire as well as six additional questions relating beliefs about imaging, physical activity and medication. Surveys were mailed to 3000 households in July–August 2018 and responses collected until September 30th, 2018.

**Results:**

Fout hundred twenty-eight surveys were returned (mean age 55 years (SD 14.6), 66% female, 90% had experienced an episode of LBP). The mean Back Beliefs Questionnaire score was 27.3 (SD 7.2), suggesting that people perceive back pain to have inevitable negative consequences. Large proportions of respondents held the following beliefs that are contrary to best available evidence: (i) having back pain means you will always have weakness in your back (49.3%), (ii) it will get progressively worse (48.0%), (iii) resting is good (41.4%) and (iv) x-rays or scans are necessary to get the best medical care for LBP (54.2%).

**Conclusions:**

A high proportion of the public believe LBP to have inevitable negative consequences and hold incorrect beliefs about diagnosis and management options, which is similar to findings from other countries. This presents challenges for clinicians and suggests that considering how to influence beliefs about LBP in the broader community could have value. Given the high prevalence of LBP and that many will consult a range of healthcare professionals, future efforts could consider using broad reaching public health campaigns that target patients, policy makers and all relevant health providers with specific content to change commonly held unhelpful beliefs.

**Supplementary Information:**

The online version contains supplementary material available at 10.1186/s12889-021-10664-5.

## Background

Low back pain (LBP) is a global health issue and a significant cause of disability. The age-standardized global prevalence of low back pain in 2019 was 70 per 1000 population [1]. It is also the leading condition contributing to the need for rehabilitative care in 134 of 204 countries [2]. In Canada, LBP is a substantial economic and social burden. Its costs are both direct (e.g., it is one of the most common reasons for seeking health care) [3] and indirect (related to time off work, lost wages and out-of-pocket expenses) [4, 5].

In the past, care for LBP involved the use of routine imaging and medication prescription and included advice to rest and avoid physical activity [6]. However, we now know that these management strategies are ineffective. Research has shown that too much rest is associated with slower recovery, medication is often minimally helpful for pain management and that activity should be promoted to improve recovery [7–9]. Additionally, we know that imaging is not helpful for diagnosis in most cases of back pain and may lead to worse outcome for some patients [10]. Based on this research, there has been consensus for the last 20 years on clinical practice guidelines for the management of LBP [11]. Specifically, clinical practice guidelines recommend that LBP treatment and management should include universal provision of information and advice to remain active and avoid excessive rest, limit the use of opioids for pain management and only use imaging in the small proportion of people with suspected specific serious pathology (e.g. cancer, infection, cauda equina or a severe nerve root compression that is unresponsive to conservative management) [11].

Recent evidence has shown that the care many patients receive from family practice and emergency departments for their LBP is divergent from clinical practice guidelines; less than 20% of patients receive evidence-based information and advice and over-prescription of imaging and opioids is common [12]. Although evidence for what is driving many of these behaviours is sparse, a recent systematic review of qualitative studies found that overuse of imaging may be in part due to patient beliefs and expectations [13]. For example, GPs reported that their patients believe an image will provide the best diagnosis for their back pain and it is easier to order an image than to try and explain otherwise [13]. The beliefs individuals hold about the causes of their back pain and about the pain itself can influence their clinical outcomes [14]. This is particularly the case for expectations about recovery and appropriate physical activity. Beliefs also impact on people’s choice of healthcare professional and preference for treatment [15]. Therefore, understanding the nature of patient beliefs and expectations regarding treatment will help coordinate efforts to improve consistency and quality of care.

## Methods

### Aim

This study aims to describe the beliefs held by the public about the assessment, management and prognosis of low back pain.

### Study design

A cross-sectional population-based survey was distributed to residents of Newfoundland to assess beliefs about low back pain. Eligible participants were residents of Newfoundland aged 18 years and older. Ethics approval was obtained from the Health Research Ethics Authority (HREA Reference # 2018.033).

### Procedure

All residents of Newfoundland formed the study population. A random sample of 380 adults from the population was required to be 95% confident in the data with a 5% margin of error (calculated using an online sample size calculator [16]). Based on response rates of previous postal surveys of 10–20%, we chose a sample of 3000 adults in Newfoundland to achieve our target sample of 380. Surveys were mailed in July to August 2018 via Canada Post Admail service. We worked with Canada Post to select routes that would reach a representative sample of Newfoundland residents. This involved first dividing the total sample size to be reflective of the population size in the 3 health districts; Eastern Health, Central Health and Western Health. Within each health district routes that targeted major urban and rural sites were identified, where multiple routes were identified in an area, one was selected at random. In this way, we attempted to ensure we reached a representative sample of all adults residing in both rural and urban sites across all 3 health districts in Newfoundland. Responses were collected until September 30th, 2018. Survey responses were entered into a SPSS file for analysis. To encourage responses, we used an incentive; this involved providing a separate reply card (postage pre-paid) with each survey. Participants could post the anonymous reply card separate to the survey, and once received by the research team, it was entered into a draw for chance to win one of three fifty-dollar gift cards.

### Survey design

The survey was written in English and included questions about participant demographics (age, gender), low back pain characteristics (previous pain or current pain; pain intensity; previous self-management treatments; previous health professionals seen; previous treatment advice from health professionals regarding rest, activity, work, medication, imaging or specialist services), and The Back Beliefs Questionnaire (BBQ) [17–19]. The BBQ was developed by Symonds et al. [17] in 1996 and was designed to measure an individual’s beliefs about the inevitable negative consequences of LBP or back trouble [17]. It has 14 items and can be used whether or not the responder has a history of LBP. Each of the 14 items is scored on 5-point Likert scales from 1 = completely disagree to 5 completely agree. The BBQ has demonstrated adequate internal consistency (Cronbach a = between 0.7 and 0.81 [17, 18];) and test-retest reliability (intraclass correlation coefficient 0.87 [17];). In addition, we also included items on specific beliefs that are relevant to the assessment and management of low back pain according to the most recent guidelines (e.g. imaging, medication, activity and rest). For beliefs about imaging, we used two items from a study by Jenkins et al. [19], and for beliefs about activity, rest and use of painkillers we used 4 items from a study by Gross et al. [20] These additional 6 items were scored on the same 5-point Likert scale as the BBQ. A copy of the survey is included in a supplementary file.

### Data analysis

Survey responses were double entered into SPSS [21] and checked for errors prior to analysis. Demographics and LBP characteristics of the sample were reported using descriptive statistics. The BBQ items and the six additional items on beliefs related to physical activity, rest and the use imaging and pain killers were presented as proportions trichotomized into disagree (disagree or strongly disagree), agree (agree or strongly agree) and unsure (neither agree or disagree). The total score for the BBQ was calculated according to the scoring method by Symonds et al. [17] (reverse scores for only 9 of the 14 items (items 1,2,3,6,8,10,12,13,14)) to provide a total score from 9 to 45. Five of the items are distractor items and are not included in the total score. Lower scores represent more negative attitudes and beliefs about back pain [17]. We decided not to impute data because this is a descriptive study and we did not conduct inferential statistics. Further, missing data rates were low across all the survey questions; hence, imputation would make little difference to the study findings.

### Patient and public involvement

Patients were involved in the decision to prioritise this research question, reviewed the survey for data collection and will be involved in the interpretation and dissemination of the results.

## Results

### Demographics

Four hundred twenty-eight surveys were returned (14% response rate). The average age of the sample was 55 years (SD 14.6 years) and 65.6% of respondents were female. Three-hundred and eight-six (90.2%) respondents reported a history of low back pain, most of whom (*n* = 339, 88.8%) had experienced low back pain in the past year. Among those with low back pain in the past year, 252 (74.3%) reported low back pain within the last week (Table 1).

**Table 1:** Demographics, LBP characteristics and outcomes of survey participants (total *n* = 428)

Item	Response	# missing
Age (Mean, SD)	55.35 (14.67)	0
Female (N, %)	281 (65.6.0)	2
**Back Pain Characteristics**		
History of low back pain (N, %)	386 (90.2)	0
Low back pain in the last 12 months (N, %) ^a^	339 (87.8)	4
Low back pain in the last week (N, %) ^a^	252 (74.3)	4
Last episode of low back pain severity [0–10] (mean, SD) ^a^	5.8 (2.3)	5
Care seeking for low back pain (N, %) ^a^	200 (51.8)	3

^a^these questions were only answered by the sample of people who reported a history of back pain (*n* = 386), the percentage is calculated from completed responses only

### Treatment and care seeking behaviours among those with a self-reported history of LBP (*n* = 386)

When asked about what they did for their back pain, 191 (49.5%) people took painkillers, 179 (46.4%) reported they rested or avoided activity, 142 (36.8%) reported other types of activities (e.g. heating pad or ice pack, stretching, or massage therapy), 109 (28.2%) did some physical activity, 56 (14.5%) reported that they went to bed or lay down, 24 (6.2%) took time off work, and 40 (10.4%) did nothing. Two-hundred (51.8%) sought medical or professional help with their back pain. Among these 200 people, most saw a family doctor 144 (72.0%); other professionals reported include chiropractor 77 (38.5%), physiotherapist 61 (30.5%), massage therapist 56 (28.0%), medical specialist 26 (13%) or pharmacist 11 (5.5%). Twenty-three (11.5%) reported ‘other’ and listed a variety of other health providers (e.g., acupuncturist, homeopathic doctor, orthopaedic surgeon, emergency doctor or osteopath). Among those who sought medical/professional help, 104 (52.0%) were advised to take painkillers, 97 (48.5%) were advised to stay active, 84 (42.0%) received referral for imaging, 59 (29.5%) were advised to rest or avoid activity, 27 (13.5%) were referred to a specialist, 21 (10.5%) were advised to take time off work and 8 (4.0%) were advised to go to bed or lay down. Sixty-seven (33.5%) reported receiving other types of advice including exercising, stretching, losing weight and using hot or cold packs.

### Back beliefs questionnaire

The mean Back Beliefs Questionnaire score for the cohort was 27.3 (SD = 7.2), indicating that our population sample believed that back pain has inevitable negative consequences. Across the 9 items, approximately 25% neither agreed nor disagreed with the belief statements. Forty percent held negative beliefs that are contrary to evidence-based management of LBP: (i) having back pain means you will always have weakness in your back, (ii) it will get progressively worse, and (iii) that resting is good. Please see Table 2 for complete breakdown of responses on the Back Beliefs Questionnaire.

**Table 2:** The Back Beliefs Questionnaire (9 items for scoring)

BBQ item	# of respondents	Disagree (1 &2) n (%)	Neutral (3) n (%)	Agree (4&5) n (%)
1. There is no real treatment for back trouble (item1)	420	245 (58.3)	110 (26.2)	65 (15.5)
2. Back trouble will eventually stop you from working (item 2)	417	172 (41.2)	103 (24.7)	142 (34.1)
3. Back trouble means periods of pain for the rest of one’s life (item 3)	416	121 (29.1)	103 (24.8)	192 (46.2)
4. Back trouble makes everything in life worse (item 6)	417	104 (24.9)	129 (30.9)	184 (44.1)
5. Back trouble means you end up in a wheelchair (item 8)	421	253 (60.1)	101 (24.0)	67 (15.9)
6. Back trouble means long periods of time off work (item 10)	414	211 (51.0)	119 (28.7)	84 (20.3)
7. Once you have had back trouble there is always a weakness (item 12)	418	122 (29.2)	90 (21.5)	206 (49.3)
8. Back trouble must be rested (item 13)	415	89 (21.4)	154 (37.1)	172 (41.4)
9. Later in life back trouble gets progressively worse (item 14)	417	94 (22.5)	123 (29.5)	200 (48.0)

### Beliefs about activity, rest and the use of imaging and pain killers

While just over half of respondents agreed (n = 230, 55.2%) that if they had back pain they should try to stay active, many also agreed (*n* = 100, 23.9%) or were unsure (*n* = 160, 38.2%) that they should rest until they got better which was similar to beliefs about going to work (*n* = 113, 26.8% agreed and *n* = 144, 34.1% were unsure). In terms of analgesics, about half of respondents (*n* = X, 47.8%) did not think that simple painkillers were enough to control most back pain. In terms of imaging, 227 (54.2%) thought that x-rays or scans are necessary to get the best medical care for LBP, and 106 (25.3%) were unsure. Similarly, 209 (50.2%) thought that everyone with LBP should have an image and 99 (23.8%). Please see Table 3 for a detailed breakdown of responses regarding beliefs about activity, rest, and the use of imaging and painkillers.

**Table 3:** Additional beliefs about activity, rest, and the use of imaging and pain killers

Belief statements	# of respondents	Disagree (1 & 2) n (%)	Neutral (3) n (%)	Agree (4 &5) n (%)
1. X-rays or scans are necessary to get the best medical care for low back pain	419	86 (20.5)	106 (25.3)	227 (54.2)
2. Everyone with low back pain should have spine imaging (e.g X-ray, CT, MRI)	416	108 (26.0)	99 (23.8)	209 (50.2)
3. If you have back pain, you should rest until it gets better	419	159 (37.9)	160 (38.2)	100 (23.9)
4. If you have back pain, you should try to stay active	417	46 (11.0) bad	141 (33.8)	230 (55.2)
5. Simple painkillers are usually enough to control most back pain	418	200 (47.8) bad	118 (28.2)	100 (23.9)
6. Most back pain settles quickly, and you can get on with normal activities such as going to work	422	165 (39.1) bad	144 (34.1)	113 (26.8)

While just over half of respondents agreed (*n* = 230, 55.2%) that if they had back pain they should try to stay active, many also agreed (*n* = 100, 23.9%) or were unsure (*n* = 160, 38.2%) that they should rest until they got better which was similar to beliefs about going to work (*n* = 113, 26.8% agreed and *n* = 144, 34.1% were unsure). In terms of analgesics, about half of respondents (*n* = 200, 47.8%) did not think that simple painkillers were enough to control most back pain. In terms of imaging, 227 (54%) thought that x-rays or scans are necessary to get the best medical care for LBP, and 106 (25%) were unsure. Similarly, 209 (50%) thought that everyone with LBP should have an image and 99 (24%) were unsure. Overall, it appears that about 50% of respondents hold beliefs that are contrary to evidence-based management regarding the use of imaging with another 20% being unsure. In terms of resting or remaining active and going to work and taking pain killers, a large proportion (approximately 35%) were unsure about the best course of action. Please see Table 3.

## Discussion

### Main findings

This study provides an overview of the beliefs about back pain among a sample of the general public living in Newfoundland, Canada. The majority of people in Newfoundland believe that back pain has inevitable negative consequences and hold beliefs about the nature, prognosis and appropriate management of LBP that are contrary to evidence-based practice. For example, we found that over 70% of people believe (or are unsure) that having an episode of back pain means that there will always be weakness in your back, it should be rested and will get progressively worse. Large proportions were also unsure or disagreed with the statements that they should try to stay active or get on with normal activities and believed (or were unsure) that imaging is necessary for the best medical care.

### Strengths and limitations

The main strength of this study was the sampling strategy that accessed a representative cross-section of the population, large enough to provide precise estimates of the factors of interest. We also used a validated questionnaire to assess beliefs about back pain and used an anonymous data collection procedure to promote truthful responses. However, the response rate was low (14%), as per other general population surveys. It is likely that since a large proportion of our respondents had had a previous episode of back pain, that this experience with back pain and its consequences may be different than the population on average. It is not known whether this experience would result in different beliefs about the condition and appropriate management and we were unable to complete an analysis of non-response bias. Another limitation is that we did not publish a protocol for this study.

### Findings in relation to literature

A recent systematic review identified 12 general population studies from Australia, Canada and the UK (total *n* = 13,319) that explored beliefs about back pain and pain management using the Back Pain Beliefs Questionnaire (BBQ) [22]. Eight of the 12 studies found that respondents agreed, on average, with beliefs that back pain has inevitable negative consequences (mean score of 27 or less on the BBQ). The results from our survey align with these findings (mean BBQ score = 27.3; SD = 7.2), suggesting that the general population in Newfoundland, Canada, hold similar beliefs to those from other countries.

In terms of beliefs about LBP imaging, a survey of 300 patients with LBP in Australia reported almost identical findings to ours. Just over half the sample believed that imaging was necessary to get the best medical care (30% were unsure) and 48% believed that everyone should have an image for their LBP (28.7% were unsure). The same study also reported comparable scores on the BBQ to our sample; mean score of 28.1 (SD = 6.8). Although they specifically surveyed patients with LBP, it is unsurprising that our findings are similar given that 90% of our survey respondents had experienced LBP, 79% in the last 12 months. Our findings were also comparable to those reported in surveys conducted in Alberta 2005 (baseline) and Saskatchewan 2005, 2006, 2007 and 2008 (controls) on all items that related to remaining active or returning to work [23]. These Canadian studies also reported comparable mean BBQ scores (range: 25.5 to 26.7). These samples also included primarily people who had experienced back pain previously (> 80%).

The BBQ includes individual items focused on the place of physical activity and rest in management of back pain. Guidelines consistently recommend the former and discourage the latter. Approximately 55% of our sample agreed that people with back pain should stay active, which was similar to most of the estimates from previous studies which fell between 55 and 65% [22]. Approximately 41% of our sample believed that people with back pain needed rest which fell in the middle of the range of estimates from previous studies of 25 to 70% [22]. Several items in the BBQ refer to expectations about prognosis or course of symptoms. Data from our sample showed that 20 to 50% of people thought back pain would have long-term, negative consequences such as long periods off work, caused permanent weakness in the back and symptoms would become progressively worse later in life. These data are comparable with a survey of Norwegians in which only 20–30% of respondents believed that back pain resolved itself over a period of weeks [24].

### Implications

The findings of this survey point to challenges for clinicians in the management of their patients with back pain, and align with findings from a clinical sample in the same jurisdiction [25]. These challenges lie with commonly held beliefs that run contrary to evidence-based management of LBP. Most notably, substantial proportions of people believe that they should stop working and rest, that their backs will forever be weak, that there is no real treatment and that imaging is necessary. This highlights the need for clinicians to engage in clear and persuasive conversations about the nature of LBP and its management. Without these conversations, evidence-based treatment recommendations such as avoiding rest, returning to work in some capacity and engaging in exercise may not make sense to patients.

### Future research

At a population level, the high prevalence of LBP means that even if a relatively low proportion of individuals hold an unhelpful belief about LBP, this translates to large numbers of people across society. Given this, there may be value in considering how to influence beliefs of the broader community i.e. beyond just treatment of those who present for care. This has been previously attempted in several mass media campaigns with mixed success, [23, 26–29] which suggests that traditional health promotion approaches may not be sufficient to change these beliefs on a broad scale. This may be partly due to a failure of traditional health promotion approaches to reach all relevant stakeholders involved in the management of LBP.

Many people with LBP will seek medical advice or care and will therefore, come into contact with a healthcare professional. Unfortunately, global evidence suggests that many health professionals who care for people with LBP have misconceptions around the causes, prognosis and treatment of LBP. For this reason, Buchbinder describes LBP as partly iatrogenic, i.e. exacerbated by medical intervention [30]. This is evidenced in our results with less than half of our respondents being given advice to remain active by their healthcare provider. Therefore, any future effort to change the general public’s attitudes, beliefs and behaviours relating to LBP must include healthcare providers and policy makers, as well as people living with LBP [31]. Comprehensive public health campaigns that target all key stakeholders, that use promising new technologies, such as personalised marketing, social networks, and digital communications, could provide a promising avenue to explore in this field.

## Conclusion

From our total sample, 90% reported experiencing back pain at some point, highlighting the prevalence of this condition in the community. We found that a majority of respondents held beliefs that were contrary to evidence-based management of LBP including that they should stop working and rest, that their backs will forever be weak, that there is no real treatment, and that imaging is necessary. This presents challenges for clinicians in the management of LBP. Given that many people with LBP will seek medical advice or care, future efforts to change the beliefs and behaviours relating to LBP must include healthcare providers and policy makers, as well as members of the public.

## Supplementary Information

**Additional file 1.**

## Data Availability

The datasets used and/or analysed during the current study are available from the corresponding author on reasonable request.

## Abbreviation

LBP: Low back pain
