# Supplementary material for: What do the general public believe about the causes, prognosis and best management strategies for low back pain? A cross-sectional study
Source: BMC Public Health. 2021 Apr 8;21:682. doi: 10.1186/s12889-021-10664-5 (PMC8028215; doi:10.1186/s12889-021-10664-5)
Supplement: Supplementary file 1 — Additional file 1. [file 12889_2021_10664_MOESM1_ESM.pdf]

Lower Back Pain Survey

Thank you for agreeing to participate in this survey. Your input is very important. The researchers are trying to find out what **people think about lower back pain**. We would like to know your general views towards lower back pain, even if you have never had back pain.

The survey includes questions about your opinions and any experience you may have had related to lower back pain, as well as your gender and age. All answers are anonymous and confidential. If there are any particular questions you do not wish to answer, please skip to the next question.

There are four pages in this survey, which should take approximately 5–10 minutes to complete. When you are finished, please return it using the postage-paid return envelope. Please return your incentive entry card *separately*.

You must be 18 years of age to participate in the survey.

1. **What is your age?_______**

2. **With which gender do you identify? _________________**

3. **Have you ever had lower back pain?**

- ❑ Don't know
- ❑ No

} ***Skip ahead to question 11 on page 3***

- ❑ Yes ***Continue to question 4***

4. **Have you had lower back pain within the last 12 months?**

- ❑ Don't know
- ❑ No

} ***Skip ahead to question 6***

- ❑ Yes ***Continue to question 5***

5. **Have you had lower back pain in the last week?**

- ❑ Don't know
- ❑ No
- ❑ Yes

6. **On a scale from 0 to 10 (where 0 is no pain and 10 is unbearable pain, the worst pain you can imagine), how would you rate the severity of your last lower back pain episode?**

❍	❍	❍	❍	❍	❍	❍	❍	❍	❍	❍
0<br>no<br>pain	1	2	3	4	5	6	7	8	9	10<br>unbearable<br>pain

7. **Thinking about the last time you experienced lower back pain, what did you do about it? (Please check all that apply.)**

- ❑ Nothing
- ❑ Rested or avoided activity
- ❑ Took time off work
- ❑ Took painkillers
- ❑ Bed rest/went to bed
- ❑ Physical activity/exercise
- ❑ Other (please specify): ____

8. **Did you seek medical/professional advice or help?**

- ❑ No *Skip ahead to question 11 on the next page*
- ❑ Yes *Continue to question 9*

9. **From whom did you seek professional advice or help? (Please check all that apply.)**

- ❑ Family doctor/GP
- ❑ Medical specialist
- ❑ Chiropractor
- ❑ Physiotherapist
- ❑ Pharmacist
- ❑ Massage therapist
- ❑ Other (please specify): ______________________________

10. **What were you advised to do by the professional(s)? (Please check all that apply.)**

- ❑ Take painkillers
- ❑ Stay active
- ❑ Rest/avoid activity
- ❑ Go to bed
- ❑ Take time off work
- ❑ See a specialist
- ❑ Other (please specify): ______________________________

11. Have you heard/viewed anything about lower back pain in the media in the past month?

- ❑ No, definitely have not heard anything
- ❑ Not sure whether I have heard anything

} ***Skip ahead to question 14 on the next page***

- ❑ Yes — ***Continue to question 12***

12. Where did you hear/view about lower back pain in the media in the past month? (Please check all that apply)

- ❑ On the radio
- ❑ In a newspaper
- ❑ On a poster
- ❑ On TV
- ❑ Can't remember where
- ❑ Other (please specify): ______________________________

13. Did the information in the media influence your beliefs about lower back pain or change how you will handle an episode of lower back pain in the future?

- ❑ No
- ❑ Yes

14. **Please read each of the following statements and indicate whether you disagree or agree with each statement on a scale of 1 to 5, where 1 is completely disagree and 5 is completely agree (keep in mind this survey is specifically focused on lower back pain):**

		completely disagree				completely agree
		1	2	3	4	5
a.	There is no real treatment for back trouble.	❍	❍	❍	❍	❍
b.	Back trouble will eventually stop you from working.	❍	❍	❍	❍	❍
c.	Back trouble means periods of pain for the rest of your life.	❍	❍	❍	❍	❍
d.	Doctors cannot do anything for back trouble.	❍	❍	❍	❍	❍
e.	A bad back should be exercised.	❍	❍	❍	❍	❍
f.	Back trouble makes everything in life worse.	❍	❍	❍	❍	❍
g.	Surgery is the most effective way to treat back trouble.	❍	❍	❍	❍	❍
h.	Back trouble may mean you end up in a wheelchair.	❍	❍	❍	❍	❍
i.	Alternative treatments are the answer to back trouble.	❍	❍	❍	❍	❍
j.	Back trouble means long periods of time off work.	❍	❍	❍	❍	❍
k.	Medication is the *only* way of relieving back trouble.	❍	❍	❍	❍	❍
l.	Once you have had back trouble there is *always* a weakness.	❍	❍	❍	❍	❍
m.	Back trouble *must* be rested.	❍	❍	❍	❍	❍
n.	Later in life back trouble gets progressively worse.	❍	❍	❍	❍	❍
o.	If you have back pain, you should rest until it gets better.	❍	❍	❍	❍	❍
p.	If you have back pain, you should try to stay active.	❍	❍	❍	❍	❍
q.	Simple painkillers are usually enough to control most back pain.	❍	❍	❍	❍	❍
r.	Most back pain settles quickly, and you can get on with normal activities such as going to work.	❍	❍	❍	❍	❍

Thank you for taking the time to complete our survey!

If you would like to enter the draw, don't forget to separately return your postage-paid return card.
